# Spinal manipulative therapy and exercise for older adults with chronic low back pain: a randomized clinical trial

**DOI:** 10.1186/s12998-019-0243-1

**Published:** 2019-05-15

**Authors:** Craig Schulz, Roni Evans, Michele Maiers, Karen Schulz, Brent Leininger, Gert Bronfort

**Affiliations:** 1 University of Minnesota, Mayo Building C504, 420 Delaware Street SE, Minneapolis, MN 55455 USA; 2 Northwestern Health Sciences University, 2501 W. 84th Street, Bloomington, MN 55431 USA; 3 Hennepin Healthcare Research Institute, 914 South 8th Street S3.116, Minneapolis, MN 55404 USA

## Abstract

**Background:**

Low back pain (LBP) is a common disabling condition in older adults which often limits physical function and diminishes quality of life. Two clinical trials in older adults have shown spinal manipulative therapy (SMT) results in similar or small improvements relative to medical care; however, the effectiveness of adding SMT or rehabilitative exercise to home exercise is unclear.

**Methods:**

We conducted a randomized clinical trial assessing the comparative effectiveness of adding SMT or supervised rehabilitative exercise to home exercise in adults 65 or older with sub-acute or chronic LBP. Treatments were provided over 12-weeks and self-report outcomes were collected at 4, 12, 26, and 52 weeks. The primary outcome was pain severity. Secondary outcomes included back disability, health status, medication use, satisfaction with care, and global improvement. Linear mixed models were used to analyze outcomes. The primary analysis included longitudinal outcomes in the short (week 4–12) and long-term (week 4–52). An omnibus test assessing differences across all groups over the year was used to control for multiplicity. Secondary analyses included outcomes at each time point and responder analyses. This study was funded by the US Department of Health and Human Services, Health Resources and Services Administration.

**Results:**

241 participants were randomized and 230 (95%) provided complete primary outcome data. The primary analysis showed group differences in pain over the one-year were small and not statistically significant. Pain severity was reduced by 30 to 40% after treatment in all 3 groups with the largest difference (eight percentage points) favoring SMT and home exercise over home exercise alone. Group differences at other time points ranged from 0 to 6 percentage points with no consistent pattern favoring one treatment. One-year post-treatment pain reductions diminished in all three groups. Secondary self-report outcomes followed a similar pattern with no important group differences, except satisfaction with care, where the two combination groups were consistently superior to home exercise alone.

**Conclusions:**

Adding spinal manipulation or supervised rehabilitative exercise to home exercise alone does not appear to improve pain or disability in the short- or long-term for older adults with chronic low back pain, but did enhance satisfaction with care.

**Trial registration:**

NCT00269321.

## Background

The world’s population is rapidly aging, with the number of individuals 60 years of age and older projected to increase three-fold by 2050 [1]. It is estimated that as many as 76 to 82% of community-dwelling older adults experience persistent non-cancer pain [2, 3]. Low back pain (LBP) is one of the leading causes of musculoskeletal related disability for older adults [4, 5] with prevalence rates ranging from 32 to 58% [6, 7]. It is estimated that 25% of older adults take analgesic medications and 8 to 14% take opioids, with use more prevalent in those with lower socioeconomic status [3, 8]. Importantly, opioid misuse among older pain sufferers is on the rise [9] while concerns continue to grow regarding the undertreatment of pain [10]. When considered in aggregate, these issues support the need to identify safe and effective non-pharmacologic pain management strategies for older adults.

Recent systematic reviews have concluded there is evidence to support the use of spinal manipulative therapy (SMT) and exercise as non-pharmacologic treatment options for chronic LBP [11, 12]. Further, interventions that promote self-management are also advocated [13] to help patients learn and engage in pain management behaviors over the long term in their daily lives [14]. Importantly, there has been relatively little research assessing the effectiveness of these approaches for older adults. A systematic review of manual therapies for older adults found moderate evidence to support their use, but noted more rigorous trials are warranted [15]. Two clinical trials in older adults have shown SMT results in similar or small improvements in function relative to medical care [16, 17]. Finally, in another study focused on older adults with neck pain, the addition of SMT to a home exercise program aimed at encouraging self-management, resulted in greater neck pain reduction at the end of 12 weeks compared to either a supervised exercise program with home exercise, or home exercise alone [18]. Currently, for older adults with LBP, it is unclear whether adding SMT or supervised rehabilitative exercise to a home exercise program would offer similar advantages as observed for neck pain.

The purpose of this randomized clinical trial was to assess the relative short- and long-term effectiveness of adding spinal manipulative therapy (SMT) or a supervised exercise program (SEP), to a home exercise program (HEP), compared to HEP alone, for adults 65 years of age and older with low back pain.

## Methods

A detailed description of the study protocol was published previously [19]. The study was funded by the U.S. Department of Health and Human Services and registered at clinicaltrials.gov (NCT00269321). This study used a parallel-group randomized controlled trial design. Institutional Review Boards at participating institutions (Northwestern Health Sciences University, # 1–15–02-04 and Minneapolis Medical Research Foundation, #04–2321) approved the study protocol, and all participants provided written consent.

### Setting and participants

The trial was conducted at Northwestern Health Sciences University (Minneapolis, Minnesota). Participants were recruited through newspaper advertisements, direct mail, and community posters. Interested individuals were initially screened by telephone interview, followed by two in-person baseline evaluation visits.

#### Inclusion criteria

Age 65 years or older; independently ambulatory, community dwelling, on a stable pain medication plan (if taken), and score of a minimum of 20 points on the Folstein Mini-Mental State Examination; primary complaint of sub-acute or chronic mechanical low back pain (≥ 6 weeks duration) with no identifiable etiology, but reproduced by movement or provocation tests; self-reported LBP baseline pain intensity rating ≥ 3 on a 0 to 10 numerical rating scale; clinical presentation of LBP meeting Quebec Task Force categories of 1, 2, 3, or 4, which includes individuals with back pain, stiffness or tenderness, with or without radiation or neurological signs.

#### Exclusion criteria

Participants were excluded if they had referred back pain from an extremity joint or viscera, suffered from significant infectious disease, were currently receiving ongoing treatment for LBP, or had any contraindications to exercise or SMT.

### Randomization

As individuals became eligible, study staff masked to upcoming treatment assignments used sequentially numbered opaque envelopes prepared by an independent study statistician to allocate participants to an intervention group. The blocked randomization scheme used a 1:1:1 allocation ratio with randomly permuted block sizes that were concealed from the study team to ensure they were masked to the sequence of treatment assignments.

### Interventions

All participants received 12 weeks of care in one of three treatment groups: 1) Home Exercise Program (HEP); 2) Supervised Exercise (SEP) + HEP; or 3) Spinal Manipulative Therapy (SMT) + HEP. Individuals were asked to refrain from seeking any additional treatment for their back pain during the treatment period. The three treatment programs are described in detail in Table 1, and in a previous publication [19]. The HEP and SEP programs were delivered by 9 exercise therapists and 2 chiropractors, and the SMT was delivered by 11 licensed chiropractors with a minimum of 5 years practice experience. All providers underwent training and certification with study investigators to standardize delivery. Intervention activities were documented on standardized forms and monitored for compliance.

**Table 1:** The study treatment programs

Intervention	Home Exercise Program (HEP)	Supervised Exercise Program (SEP) + HEP	Spinal Manipulative Therapy (SMT) + HEP
Components	Information and instructions for self-care for pain (postural adjustments during activities of daily living; use of ice, heat, medications; importance of movement and staying active). Instructions in low load exercises with graded progressions, to be done at home to improve balance and coordination, trunk strength and endurance. Stretching exercises (seated or standing lumbar flexion, full spine flexion/extension motion cycles, quadriceps stretch, hamstring stretch, hip stretch, head retraction, and chest expansion). Muscle strength and endurance exercises: chair squats, abdominal curls, seated back extension (isometric or using resistance tubing), seated upright rows (using resistance tubing), and push-ups. Balance exercises: standing knee lifts, standing straight-leg hip flexion and extension.	Information and instructions for self-care for pain (postural adjustments during activities of daily living; use of ice, heat, medications; importance of movement and staying active). Light aerobic warm up on stationary equipment. Instructions, monitoring and encouragement in low load exercise with graded progressions, with an emphasis on high repetitions (up to 20) to increase endurance, strength and balance. Stretching, strength and balance exercises as described for HEP, with the addition of neck flexion, quadruped, lunges, side bridging, and trunk extension exercises on an adjustable angle roman chair.	Manual treatment based on physical condition and tolerance. Up to 4 min of adjunct therapies to facilitate SMT (light soft tissue massage, active and passive stretching, ischemic compression of tender points, ice and heat). High velocity, low amplitude SMT when possible. Other manual therapies if needed (low velocity, low amplitude SMT or mobilization, flexion-distraction manipulation, drop-table assisted SMT.
Design & delivery format	Tailored to individual ability. Individualized guidance by exercise therapists who closely monitored form, modified exercises, prescribed progressions, and provided encouragement. Binder with pain management tips, illustrated instructions and simple diary to record performance of exercise at home.	Tailored to individual ability. Individualized guidance by exercise therapists who closely monitored form, modified exercises, prescribed progressions, and provided encouragement. Binder with illustrated descriptions, simple diary to record performance of exercise at home.	Individualized: number of visits, spinal levels treated, SMT and manual therapy technique used and adjunct therapies determined by provider according to patient needs and tolerance.
Delivery method	One-on-one, in person --therapist lead	One-on-one, in person ----therapist lead	One-on-one, in person Licensed chiropractor
Dose	• 4 sessions • 45–60 min per session • Maximum frequency: 1 times/week	• 20 sessions • 60 min per session • Maximum frequency: 1 times/week	• Up to 20 sessions (based on discretion of provider and patient preferences) • 10 to 20 min per session • Maximum frequency: 2 times/week

### Outcome measures

**Fig. 1:**
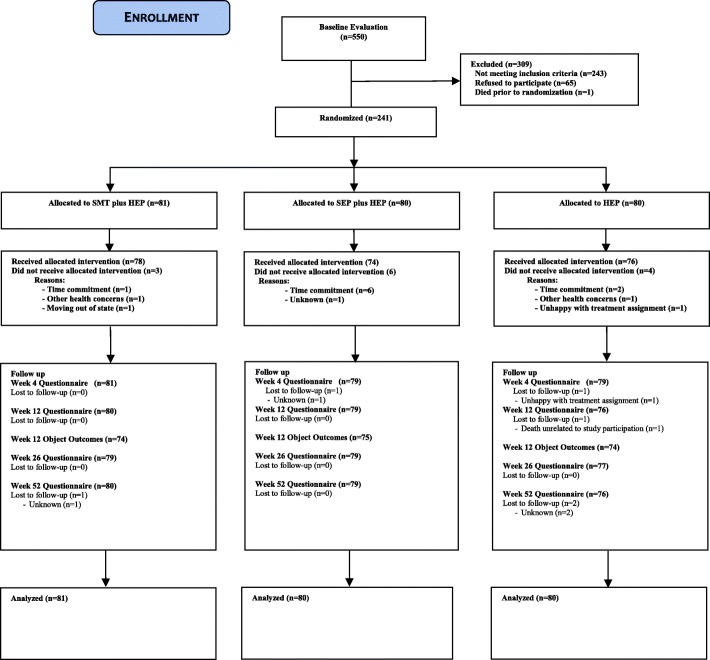
Study flow diagram. Lost to follow-up: participants who did not provide data at the specified time point and thereafter. HEP, home exercise program; SEP, supervised exercise program; SMT, spinal manipulative therapy

Outcomes were measured by patient self-report questionnaires, blinded objective assessment, and in- person and telephone interviews. Patient self-report questionnaires were collected at baseline, and 4, 12, 26, and 52 weeks post-randomization. Participant flow, study visits, and evaluations are outlined in Fig. 1.

#### Primary outcome measure

Participants rated their typical level of back pain over the last week, using an ordinal 11-box scale (0 = no pain, 10 = the worst pain possible) [20].

#### Secondary outcome measures

Low back disability was measured with the 23-item Modified Roland Scale [21, 22]. General health status was measured by the Medical Outcomes Study Short Form 36-item Health Survey (SF-36 D) [23]. Improvement was rated on a 9-point ordinal scale, with responses ranging from “100% improved” to “100% worse” [24]. Overall satisfaction with care, were rated on a 7-point scale, with responses ranging from “completely satisfied” to “completely dissatisfied” [25]. Weekly frequency of non-prescription and prescription medication use for LBP was measured on a 5-point scale. Adherence with home exercise was measured at weeks 26 and 52. Additionally, serious adverse events were recorded and classified according to relationship with treatment.

#### Objective outcome measures

Objective outcomes were measured by study staff blinded to treatment assignment at baseline and week 12 (post-intervention). Lumbar and spine dynamic motion were assessed using the Zebris CMS-HS Spine Motion Analyzer (Zebris Inc., Isny im Allgau, Germany) [26, 27]. Isometric muscle flexion and extension strength was measured by computerized digital myograph (DM2000, Myotech Corp., FL). Static muscle endurance was measured with participants in a prone neutral position unsupported from the waist up for extension and recumbent (60° angle) position for flexion [28, 29]. The “Timed Up and Go” test, was also performed [30, 31]. Hand grip strength was measured bilaterally with a hydraulic dynamometer (Jamar Hand dynamometer, Sammons Preston – U.S.A, Bolingbrook, IL) with the subjects positioned following the recommendations by the American Society of Hand Therapists [32].

#### Health care utilization

Health care utilization (within and outside of the studies) was measured using standardized clinician treatment forms (each visit, weeks 1 to 12), monthly phone call interviews (weeks 16 to 52) and patient self-report questionnaires (baseline and weeks 4, 12, 26 and 52). Productivity loss was measured through patient self-report (weeks 4, 12, 26, and 52) using three questions from the National Health Interview Survey (NHIS) [33].

#### Power calculation

Informed by results of our previous LBP trials, we were interested in powering this trial to detect a difference of 8 percentage points in the primary outcome measure of patient self-rated pain between the highest and lowest group means [34, 35]. With an alpha level of 0.05, 70 participants per group provided power of 0.88 to detect this difference. To allow for a drop-out rate of 15%, 240 participants (80 participants per group) were required.

### Statistical analysis

An intention-to-treat approach was used, analyzing all observed data from participants according to their randomized treatment assignment. Data analyses were performed in STATA, version 13.0 (StataCorp 2013, Stata Statistical Software: Release 13, College Station, TX; StataCorp LP). The statistician was blinded to group allocation for all analyses. The primary and secondary outcomes were analyzed using linear mixed effect models including fixed effects for time, treatment, and a time-by-treatment interaction, and a random intercept to account for within-subject correlation. The model included the baseline outcome as a covariate (when appropriate).

#### Primary outcome measure analysis

##### Primary analysis

The primary outcomes were group differences in pain severity in the short-term (weeks 4 to 12) and long-term profiles (weeks 4 to 52) from the linear mixed effect model. Before conducting the analysis, a new strategy to control for multiplicity due to the three group and repeated measure design was introduced. We used Fisher’s protected least significant difference approach [36]. An area under the curve minus baseline summary measure [37, 38] was used as the omnibus test to determine whether the long-term pain profiles (including 4, 12, 26, and 52 weeks) were different between groups. The omnibus test needed to be significant (*P* value = 0.05) for determining the significance levels of group differences in short- or long-term outcomes, otherwise only 95% confidence intervals would be presented. Clinical and demographic variables were included as covariates if they were at least moderately correlated (0.5) with change in outcomes [39]. Linear mixed effect model analyses provide unbiased estimates when data are missing at random [40]. Sensitivity analyses imputing (1) the 10th percentile and (2) the 90th percentile by group for the primary outcome at each time point were conducted to assess the potential impact of data missing not at random [41].

##### Secondary analysis

Secondary analysis of the primary outcome measure included group differences at weeks 4, 12, 26, and 52. In addition, responder analyses for no pain reduction, or pain reductions of ≥30% (meaningful improvement), ≥50% (substantial improvement [42]), ≥75, and 100% were performed at weeks 12, 26, and 52 [43]. Differences in proportions of responders between groups were calculated, and 95% confidence intervals (CIs) were analyzed using the Wilson method for risk differences [44]. Cumulative responder analysis graphs were created to display the proportion of responders for all possible levels of pain reduction [45].

##### Secondary outcome measure analysis

Secondary patient-rated outcome measures analyzed for this publication included disability, improvement, satisfaction, medication use, and quality of life. These analyses included all individual time points, the short-term profile (including 4 and 12 weeks), and the long-term profile (including 4 through 52 weeks). The same omnibus test approach used for the primary outcome was applied to the secondary outcomes to control for multiplicity. Objective outcomes analyzed included isometric strength, static endurance, handgrip strength, timed up and go, and range of motion. These analyses were conducted using linear mixed models with baseline as an outcome to assess between group differences in changes at 12 weeks [38]. Results of the qualitative data will be reported in a separate publication.

## Results

### Baseline characteristics

A summary of patient recruitment, participation, treatment adherence, and attrition during the study is shown in Fig. 1. Participants were recruited between 2004 and 2006, with follow-up data collection completed in 2007. A total of 550 individuals were evaluated for the study, of which 241 were randomized. Demographic and baseline clinical characteristics of all randomized participants are displayed in Table 2. Randomization resulted in three groups comparable on most baseline variables with four potentially important exceptions: age, gender, duration of LBP and treatment effect expectation (Table 2). None of these variables were more than weakly correlated with the primary outcome (absolute values from 0.04 to 0.23), and thus were not included as covariates in the analyses.

**Table 2:** Baseline demographics and clinical characteristics (mean [SD] unless otherwise noted)

Parameter	Treatment group		
	SMT + HEP	SEP + HEP	HEP
n	81	80	80
Age	72.5 (5.6)	73.6 (5.3)	74.7 (5.6)
Female, n (%)	46 (56.8)	38 (47.5)	40 (50.0)
Race, n (%)			
White	78 (96.3)	77 (96.3)	77 (96.3)
Asian or Pacific Islander	0 (0)	0 (0)	1 (1.3)
Black	3 (3.7)	2 (2.5)	1 (1.3)
Other	0 (0)	1 (1.3)	0 (0)
Not reported	0 (0)	0 (0)	1 (1.3)
Ethnicity, n (%)			
Hispanic	0 (0)	0 (0)	0 (0)
Education			
Less than high school	3 (3.7)	1 (1.3)	4 (5.0)
High school degree	26 (32.1)	29 (36.3)	21 (26.3)
Some college	23 (28.4)	26 (32.5)	25 (31.3)
College degree	17 (21.0)	14 (17.5)	12 (15.0)
Professional degree	12 (14.8)	10 (12.5)	18 (22.5)
Working, n (%)	11 (13.6)	18 (22.5)	16 (20.0)
BMI	29.0 (6.4)	28.3 (5.1)	28.7 (4.3)
Duration [years]	13.7 (15.7)	12.1 (15.1)	12.9 (15.8)
-Median [25th to 75th percentiles]	10.0 [1.6 to 20.0]	5.0 [1.8 to 19.5]	5.0 [2.5 to 20.0]
Chronic (current episode ≥12 weeks), n (%)	78 (96.3)	77 (96.3)	78 (97.5)
Radiation to lower extremity, n (%)	27 (33.3)	32 (40.0)	34 (42.5)
Awake at night due to back pain, n (%)	32 (39.5)	21 (26.3)	18 (22.8)
Traumatic onset, n (%)	27 (33.3)	15 (18.8)	15 (18.8)
Prior treatment in past 3 months, n (%)	24 (29.6)	24 (30.0)	20 (25.0)
History of depression or anxiety, n (%)	16 (19.8)	12 (15.0)	12 (15.0)
Neck pain, n (%)	58 (71.6)	60 (75.0)	58 (72.5)
Tobacco use, n (%)	3 (3.7)	10 (12.5)	4 (5.0)
Low back pain severity [0–10]	5.07 (1.60)	5.31 (1.45)	5.14 (1.43)
Low back disability (Roland Morris) [0–100]	45.5 (20.9)	42.9 (17.9)	45.3 (16.5)
Medication use (0–4)	1.63 (1.50)	1.68 (1.47)	2.09 (1.67)
Expectation of improvement at the end of treatment (1–9)	1.52 (0.50)	1.64 (0.56)	1.99 (0.72)

*BMI* body mass index, *HEP* home exercise program, *SEP* supervised exercise program, *SMT* spinal manipulative therapy

### Treatment frequency and adherence

Adherence to study interventions was high with 92% of the SMT + HEP group (78/81), 91% of the SEP + HEP group (74/80), and 95% of the HEP group (76/80) completing the required sessions. The mean (SD) number of SMT visits was 15.1 (4.1); the mean number of SEP sessions was 16.0 (3.9); and the mean number of HEP sessions was 3.9 (0.6) for HEP alone. The mean number of HEP sessions in the combined groups was also 3.9. Compliance with home exercise at weeks 26 and 52 was similar among the three groups with over 80% of participants reporting at least 1 day/week of home exercise (≥193/232) and over 35% of participants reporting exercising on 3 or more days/week (≥99/232). During the 12-week intervention phase, 13 participants reported additional LBP-related healthcare use, 3 from SMT + HEP, 6 from SEP + HEP, and 4 from HEP. After the intervention phase, 42 participants in the SMT + HEP group reported LBP-related healthcare use compared to 25 in the SEP + HEP group and 25 in the HEP group (Pearson chi-square = 9.7, *p*-value = 0.008). Participants with additional healthcare use tended to have more severe pain at baseline and throughout the one-year follow-up period.

### Effectiveness assessments

#### Primary analysis of the primary outcome measure

**Fig. 2:**
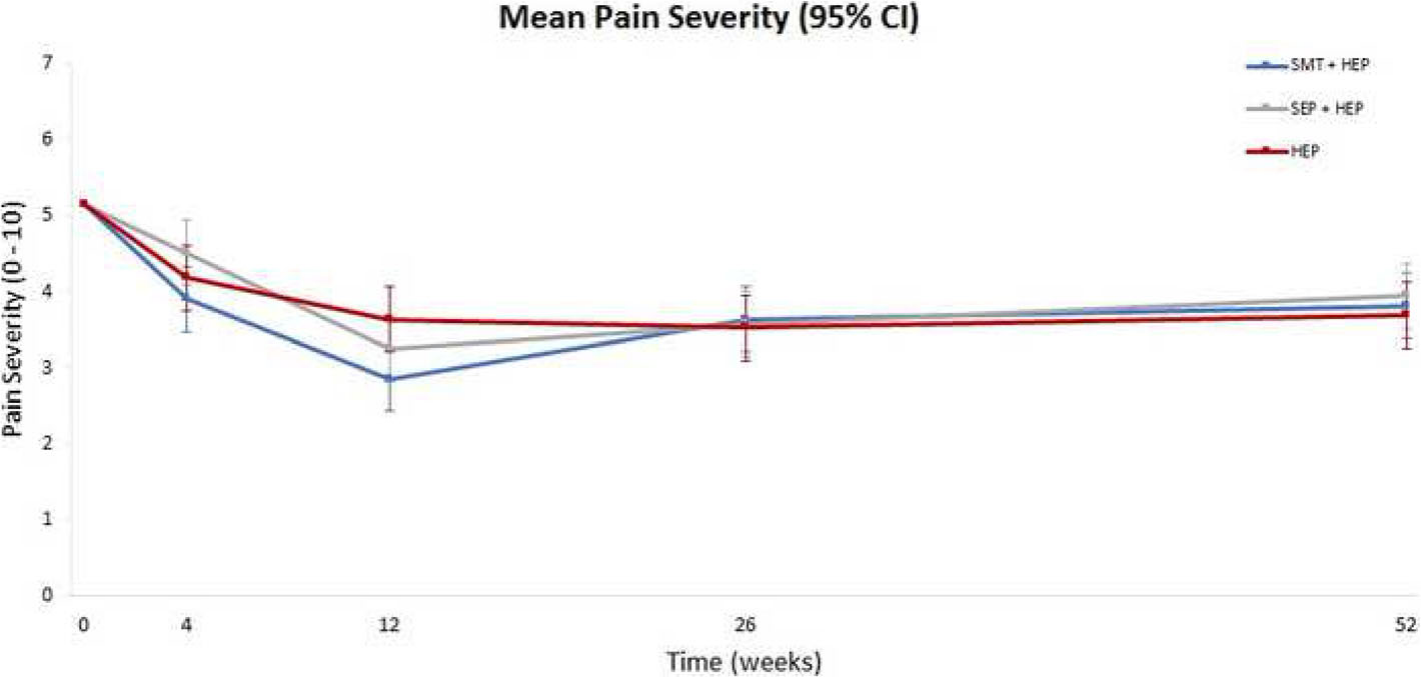
Change in mean pain severity over time. HEP, home exercise program; SEP, supervised exercise program; SMT, spinal manipulative therapy

Of the 241 participants randomized, 230 (95%) provided primary outcome data at every follow up point. Overall, there was a reduction in pain severity, the primary outcome, of approximately 30 to 40% at the end of the 12-week treatment period and approximately 25% at one year in all three groups (Fig. 2). Cumulative group differences in the long-term pain profiles (from baseline to one year) were small and not statistically significant (omnibus test for group differences: *P* = 0.76; see Table 3). Group differences in short-term pain severity profiles ranged from 0 to 5 percentage points (Table 3). Sensitivity analyses to assess the potential impact of data missing not at random resulted in group differences of similar magnitude (+/− 0.13 percentage points) and in the same direction as the primary analysis with no changes in statistical significance.

**Table 3:** Primary and Secondary Patient-rated Outcomes

	SMT + HEP	SEP + HEP	HEP	SMT + HEP minus HEP	SEP + HEP minus HEP	SMT + HEP minus SEP + HEP
Low back pain severity [0–10]						
Week 0 Mean (SD)	5.07 (1.60)	5.31 (1.45)	5.14 (1.43)			
Week 4 Mean (95% CI)	3.90 (3.48 to 4.33)	4.51 (4.08 to 4.94)	4.19(3.76 to 4.62)	−0.28 (− 0.89 to 0.32)	0.33(− 0.28 to 0.94)	− 0.61(−1.22 to − 0.01)
Week 12 Mean (95% CI)	2.85 (2.43 to 3.28)	3.25(2.82 to 3.68)	3.64(3.20 to 4.07)	− 0.78(−1.39 to − 0.17)	− 0.39 (− 1.00 to 0.22)	−0.39 (− 1.00 to 0.21)
Short term Mean (95% CI)				−0.48 (− 1.00 to 0.03)	0.04 (− 0.48 to 0.56)	− 0.53 (− 1.04 to − 0.01)
Week 26 Mean (95% CI)	3.64 (3.21 to 4.07)	3.58 (3.15 to 4.01)	3.52 (3.08 to 3.95)	0.12 (− 0.49 to 0.73)	0.06 (− 0.56 to 0.67)	0.06 (− 0.55 to 0.67)
Week 52 Mean (95% CI)	3.81 (3.39 to 4.24)	3.94 (3.51 to 4.37)	3.68 (3.25 to 4.12)	0.13 (− 0.48 to 0.74)	0.26 (− 0.35 to 0.87)	− 0.13 (− 0.74 to 0.48)
Long term Mean (95% CI)				−0.13 (− 0.59 to 0.34)	0.04 (− 0.43 to 0.52)	− 0.17 (− 0.64 to 0.30)
Omnibus test for differences in long term longitudinal profile for pain severity (weeks 4 through 52) between groups *P*-value = 0.76						
Low back disability (Roland Morris) [0–100]						
Week 0 Mean (SD)	45.5 (20.9)	42.9 (17.9)	45.3 (16.5)			
Week 4 Mean (95% CI)	36.2 (32.4 to 40.0)	34.2 (30.4 to 38.0)	34.8 (31.0 to 38.6)	1.38 (−3.99 to 6.75)	−0.60 (−6.01 to 4.81)	1.98 (− 3.39 to 7.36)
Week 12 Mean (95% CI)	29.9 (26.1 to 33.7)	25.3 (21.5 to 29.1)	30.0 (26.1 to 33.9)	−0.13 (−5.56 to 5.29)	−4.70 (−10.15 to 0.74)	4.57 (− 0.83 to 9.97)
Short term Mean (95% CI)				0.78 (−3.99 to 5.54)	−2.24 (−7.04 to 2.55)	3.02 (−1.75 to 7.78)
Week 26 Mean (95% CI)	30.0 (26.2 to 33.9)	31.1 (27.3 to 35.0)	34.8 (31.0 to 38.7)	−4.81 (−10.25 to 0.63)	−3.70 (−9.16 to 1.76)	− 1.11 (−6.55 to 4.33)
Week 52 Mean (95% CI)	34.3 (30.5 to 38.1)	33.4 (29.5 to 37.2)	32.9 (29.0 to 36.8)	1.41 (−4.04 to 6.85)	0.45 (−5.00 to 5.90)	0.96 (−4.45 to 6.36)
Long term Mean (95% CI)				−1.42 (−5.88 to 3.04)	−2.47 (−6.95 to 2.01)	1.05 (−3.40 to 5.50)
Omnibus test for differences in long term longitudinal profile for disability (weeks 4 through 52) between groups P-value = 0.56						
Improvement [1–9]						
Week 4 Mean (95% CI)	3.85 (3.55 to 4.16)	3.84 (3.52 to 4.15)	3.91 (3.6 to 4.22)	− 0.06 (− 0.5 to 0.38)	−0.08 (− 0.52 to 0.36)	0.02 (− 0.42 to 0.45)
Week 12 Mean (95% CI)	3.11 (2.80 to 3.42)	3.00 (2.69 to 3.31)	3.34 (3.02 to 3.65)	− 0.23 (− 0.67 to 0.21)	−0.34 (− 0.78 to 0.1)	0.11 (− 0.33 to 0.55)
Short term Mean (95% CI)				−0.13 (− 0.51 to 0.26)	−0.18 (− 0.57 to 0.21)	0.05 (− 0.33 to 0.44)
Week 26 Mean (95% CI)	3.43 (3.12 to 3.75)	3.38 (3.07 to 3.7)	3.54 (3.23 to 3.86)	− 0.11 (− 0.55 to 0.33)	−0.16 (− 0.6 to 0.28)	0.05 (− 0.39 to 0.49)
Week 52 Mean (95% CI)	3.72 (3.41 to 4.03)	3.54 (3.23 to 3.86)	3.64 (3.33 to 3.96)	0.07 (− 0.37 to 0.51)	−0.1 (− 0.54 to 0.34)	0.17 (− 0.27 to 0.61)
Long term Mean (95% CI)				−0.08 (− 0.45 to 0.28)	−0.17 (− 0.54 to 0.19)	0.09 (− 0.27 to 0.45)
Omnibus test for differences in long term longitudinal profile for improvement (weeks 4 through 52) between groups *P*-value = 0.65						
Satisfaction [1–7]						
Week 4 Mean (95% CI)	1.93 (1.72 to 2.14)	1.77 (1.56 to 1.98)	2.35 (2.14 to 2.57)	−0.43 (− 0.73 to − 0.13)	−0.58 (− 0.88 to − 0.28)	0.15 (− 0.15 to 0.45)
*P*-value				0.005	< 0.001	0.31
Week 12 Mean (95% CI)	1.86 (1.65 to 2.08)	1.68 (1.47 to 1.9)	2.38 (2.17 to 2.6)	−0.52 (− 0.82 to − 0.22)	−0.7 (− 1 to − 0.4)	0.18 (− 0.12 to 0.48)
*P*-value				0.001	< 0.001	0.24
Short term Mean (95% CI)				−0.46 (− 0.74 to − 0.19)	−0.63 (− 0.9 to − 0.36)	0.16 (− 0.11 to 0.44)
*P*-value				< 0.001	< 0.001	0.23
Week 26 Mean (95% CI)	2.07 (1.86 to 2.29)	1.82 (1.61 to 2.04)	2.46 (2.25 to 2.68)	−0.39 (− 0.69 to − 0.09)	−0.64 (− 0.94 to − 0.34)	0.25 (− 0.05 to 0.55)
P-value				0.012	< 0.001	0.10
Week 52 Mean (95% CI)	2.17 (1.96 to 2.38)	1.87 (1.66 to 2.09)	2.54 (2.32 to 2.75)	−0.37 (− 0.67 to − 0.06)	−0.66 (− 0.97 to − 0.36)	0.3 (− 0.003 to 0.6)
P-value				0.018	< 0.001	0.052
Long term Mean (95% CI)				−0.42 (− 0.67 to − 0.16)	−0.65 (− 0.91 to − 0.39)	0.24 (− 0.02 to 0.49)
P-value				0.002	< 0.001	0.07
Omnibus test for differences in long term longitudinal profile for satisfaction (weeks 4 through 52) between groups P-value < 0.001						
Medication use [0–4]						
Week 0 Mean (SD)	1.63 (1.50)	1.68 (1.47)	2.09 (1.67)			
Week 4 Mean (95% CI)	1.53 (1.25 to 1.81)	1.52 (1.24 to 1.81)	1.60 (1.31 to 1.88)	−0.07 (− 0.47 to 0.34)	−0.07 (− 0.48 to 0.33)	0.01 (− 0.39 to 0.41)
Week 12 Mean (95% CI)	1.51 (1.23 to 1.8)	1.28 (1 to 1.57)	1.61 (1.32 to 1.9)	−0.09 (− 0.5 to 0.32)	−0.32 (− 0.73 to 0.08)	0.23 (− 0.17 to 0.63)
Short term Mean (95% CI)				−0.08 (− 0.42 to 0.27)	−0.17 (− 0.52 to 0.17)	0.10 (− 0.24 to 0.44)
Week 26 Mean (95% CI)	1.61 (1.33 to 1.9)	1.50 (1.21 to 1.78)	1.51 (1.22 to 1.8)	0.1 (−0.3 to 0.51)	−0.01 (− 0.42 to 0.4)	0.12 (− 0.29 to 0.52)
Week 52 Mean (95% CI)	1.77 (1.49 to 2.06)	1.45 (1.16 to 1.73)	1.47 (1.18 to 1.76)	0.3 (−0.1 to 0.71)	−0.02 (− 0.43 to 0.39)	0.33 (− 0.08 to 0.73)
Long term Mean (95% CI)				0.09 (−0.22 to 0.40)	−0.09 (− 0.40 to 0.22)	0.18 (− 0.13 to 0.49)
Omnibus test for differences in long term longitudinal profile for medication use (weeks 4 through 52) between groups P-value = 0.51						
SF-36 PCS [0–100]						
Week 0 Mean (SD)	37.9 (8.7)	39.3 (7.0)	38.4 (7.3)			
Week 4 Mean (95% CI)	40.8 (39.4 to 42.1)	40.8 (39.4 to 42.1)	40.2 (38.9 to 41.5)	0.55 (−1.33 to 2.43)	0.55 (− 1.35 to 2.44)	0.00 (− 1.88 to 1.88)
Week 12 Mean (95% CI)	43.0 (41.7 to 44.4)	43.8 (42.5 to 45.2)	42.5 (41.1 to 43.8)	0.58 (− 1.32 to 2.48)	1.39 (−0.52 to 3.3)	− 0.81 (− 2.7 to 1.08)
Short term Mean (95% CI)				0.56 (−1.09 to 2.21)	0.88 (−0.77 to 2.54)	−0.32 (− 1.98 to 1.33)
Week 26 Mean (95% CI)	41.7 (40.3 to 43)	43.0 (41.7 to 44.4)	42.0 (40.6 to 43.4)	−0.34 (− 2.24 to 1.57)	1.02 (− 0.89 to 2.93)	−1.36 (− 3.26 to 0.55)
Week 52 Mean (95% CI)	40.6 (39.2 to 41.9)	42.3 (40.9 to 43.6)	42.1 (40.7 to 43.4)	−1.52 (− 3.42 to 0.39)	0.19 (− 1.72 to 2.1)	−1.70 (− 3.6 to 0.19)
Long term Mean (95% CI)				−0.34 (−1.87 to 1.2)	0.83 (− 0.71 to 2.37)	− 1.16 (− 2.7 to 0.37)
Omnibus test for differences in long term longitudinal profile for SF-36 PCS (weeks 4 through 52) between groups P-value = 0.31						
SF-36 MCS [0–100]						
Week 0 Mean (SD)	53.9 (8.4)	55.5 (8.2)	55.9 (7.4)			
Week 4 Mean (95% CI)	56.7 (55.5 to 58)	55.9 (54.6 to 57.2)	56.3 (55 to 57.6)	0.47 (−1.36 to 2.3)	−0.38 (−2.22 to 1.46)	0.85 (−0.97 to 2.68)
Week 12 Mean (95% CI)	56.6 (55.3 to 57.9)	56.1 (54.8 to 57.4)	56.5 (55.2 to 57.8)	0.07 (−1.78 to 1.92)	−0.41 (−2.26 to 1.44)	0.48 (− 1.35 to 2.32)
Short term Mean (95% CI)				0.31 (−1.24 to 1.86)	−0.39 (− 1.95 to 1.16)	0.71 (− 0.84 to 2.25)
Week 26 Mean (95% CI)	56.4 (55 to 57.7)	55.1 (53.8 to 56.4)	54.7 (53.4 to 56.1)	1.61 (−0.26 to 3.47)	0.37 (−1.49 to 2.23)	1.24 (−0.61 to 3.1)
Week 52 Mean (95% CI)	54.2 (52.9 to 55.5)	55.9 (54.6 to 57.2)	55 (53.7 to 56.4)	−0.86 (−2.72 to 1)	0.84 (−1.01 to 2.7)	−1.71 (−3.55 to 0.14)
Long term Mean (95% CI)				0.49 (−1.24 to 1.86)	0.23 (−1.18 to 1.63)	0.26 (− 1.14 to 1.66)
Omnibus test for differences in long term longitudinal profile for SF-36 MCS (weeks 4 through 52) between groups P-value = 0.79						

*CI* confidence interval, *HEP* home exercise program, *SEP* supervised exercise program, *SMT* spinal manipulative therapy

#### Secondary analysis of the primary outcome measure

The largest between group difference at any individual time point was eight percentage points favoring SMT + HEP compared to HEP alone at 12 weeks (the end of treatment). All other group differences ranged from zero to six percentage points with no consistent pattern favoring one treatment group over another.

### Responder analysis

**Fig. 3:**
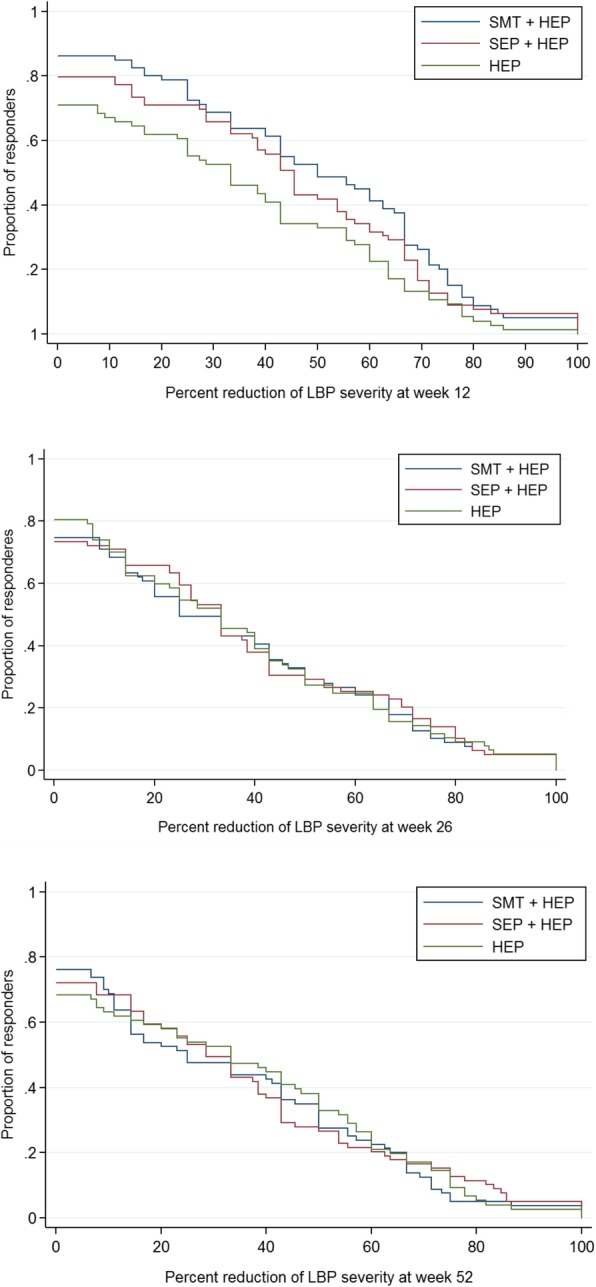
Cumulative responder graphs—the y-axis displays the proportion of participants who reported a percent reduction in pain severity from baseline equal to or greater than the value on the x-axis. HEP, home exercise program; SEP, supervised exercise program; SMT, spinal manipulative therapy

Detailed results from the responder analyses are provided in Table 4. The proportion of participants reporting reductions in pain severity across all possible thresholds is shown in Fig. 3. Differences in proportions for reduction of LBP severity were mostly below 10%. The largest differences were for SMT + HEP over HEP alone at week 12 where 16 and 18% more participants had pain severity reductions of 30 and 50%, respectively.

**Table 4:** Responder Analyses

% Pain reduction	Treatment groups			Group differences (95% CI)		
	SMT + HEP	SEP + HEP	HEP	SMT + HEP minus HEP	SEP + HEP minus HEP	SMT + HEP minus SEP + HEP
Week 12^a^						
No reduction or worsening	13.8	20.3	29.0	−15.2 (− 27.7 to 2.3)	−8.7 (− 22.0 to 4.9)	− 6.5 (− 18.2 to 5.3)
≥ 30%	68.8	65.8	52.6	16.1 (0.8 to 30.5)	13.2 (−2.2 to 27.8)	2.9 (− 11.5 to 17.2)
≥ 50%	52.5	43.0	34.2	18.3 (2.7 to 32.6)	8.8 (−6.4 to 23.5)	9.5 (−5.9 to 24.3)
≥ 75%	20.0	12.7	10.5	9.5 (−2.0 to 20.7)	2.1 (−8.4 to 12.6)	7.3 (−4.3 to 18.9)
100%	5.0	6.3	1.3	3.7 (−2.8 to 10.9)	5.0 (−1.8 to 12.7)	−1.3 (−9.6 to 6.7)
Week 26^b^						
No reduction or worsening	25.3	26.6	19.5	5.8 (−7.3 to 18.7)	7.1 (−6.2 to 20.0)	− 1.3 (− 14.8 to 12.3)
≥ 30%	49.4	53.2	52.0	−2.6 (− 17.8 to 12.8)	1.2 (−14.1 to 16.5)	−3.8 (− 18.9 to 11.5)
≥ 50%	32.9	30.4	32.5	0.4 (− 14.1 to 14.9)	−2.1 (− 16.4 to 12.3)	2.5 (− 11.8 to 16.7)
≥ 75%	12.7	16.5	14.3	−1.6 (− 12.7 to 9.3)	2.2 (−9.4 to 13.6)	− 3.8 (− 15.0 to 7.4)
100%	5.1	5.1	5.2	−0.1 (−8.2 to 7.8)	− 0.1 (− 8.2 to 7.8)	0.0 (− 7.9 to 7.9)
Week 52^c^						
No reduction or worsening	23.8	27.9	31.6	−7.8 (−21.5 to 6.1)	−3.7 (−17.8 to 10.5)	−4.1 (− 17.5 to 9.4)
≥ 30%	47.5	49.4	52.6	−5.1 (−20.3 to 10.3)	−3.3 (− 18.5 to 12.2)	− 1.9 (− 17.0 to 13.4)
≥ 50%	35.0	27.9	38.2	− 3.2 (− 17.9 to 11.7)	−10.3 (− 24.5 to 4.4)	7.2 (− 7.2 to 21.1)
≥ 75%	7.5	15.2	14.5	−7.0 (− 17.4 to 3.1)	0.7 (− 10.8 to 12.1)	− 7.7 (− 18.0 to 2.4)
100%	3.8	5.1	2.6	1.1 (− 5.8 to 8.1)	2.4 (− 4.7 to 9.9)	− 1.3 (− 9.0 to 6.1)

Percentage of participants with no reduction or worsening, 30, 50, 75%, or 100% reduction in pain severity

^a^Analysis includes 80 participants in the SMT + HEP group, 79 in the SEP + HEP group, and 76 in the HEP group

^b^Analysis includes 79 participants in the SMT + HEP group, 79 in the SEP + HEP group, and 77 in the HEP group

^c^Analysis includes 80 participants in the SMT + HEP group, 79 in the SEP + HEP group, and 76 in the HEP group

*CI* confidence interval, *HEP* home exercise program, *SEP* supervised exercise program, *SMT* spinal manipulative therapy

### Secondary outcomes

The secondary outcomes followed a similar pattern with no important group differences. The exception was satisfaction with care, where the two combination groups (SMT + HEP, SEP + HEP) were superior to home exercise alone at all time points (see Table 3). Except for a few small differences favoring SEP + HEP over SMT + HEP for flexion static endurance and hand grip strength, the objective outcomes showed the same pattern as the patient rated outcomes (see Table 5). Health care and societal cost comparisons along with cost-utility and cost-effectiveness analyses will be addressed in a separate publication.

**Table 5:** Mean change in objective measures at week 12 (95% CI)

	Treatment groups			Group differences		
	SMT + HEP	SEP + HEP	HEP	SMT + HEP minus HEP	SEP + HEP minus HEP	SMT + HEP minus SEP + HEP
Isometric strength (lbs)						
Extension	9.1 (5.0 to 12.2)	4.4 (0.3 to 8.4)	6.8 (2.7 to 10.9)	2.3 (−3.5 to 8.1)	− 2.4 (− 8.1 to 3.4)	4.7 (− 1.1 to 10.4)
Flexion	1.4 (−1.4 to 4.1)	1.0 (−1.8 to 3.8)	2.0 (− 0.8 to 4.8)	− 0.6 (− 4.5 to 3.3)	−1.0 (− 4.9 to 3.0)	0.4 (− 3.6 to 4.3)
Static endurance (sec)						
Extension	18.1 (10.0 to 26.3)	25.3 (17.2 to 33.4)	21.7 (13.5 to 29.9)	−3.6 (− 15.2 to 8.0)	3.6 (− 7.9 to 15.2)	− 7.2 (− 18.7 to 4.3)
Flexion	9.1 (− 2.0 to 20.2)	25.2 (14.2 to 36.3)	15.8 (4.7 to 27.0)	−6.7 (− 22.4 to 9.0)	9.4 (− 6.3 to 25.1)	−16.1 (− 31.8 to − 0.5)
Handgrip strength test^a^ (kg)	−0.7 (− 1.8 to 0.3)	1.1 (− 0.02 to 2.2)	0.2 (− 0.9 to 1.2)	−0.9 (− 2.4 to 0.6)	0.9 (− 0.6 to 2.4)	− 1.8 (− 3.4 to − 0.3)
Timed up and go test (sec)	−0.7 (−1.1 to − 0.2)	−0.7 (− 1.1 to − 0.2)	−0.5 (− 0.9 to − 0.03)	−0.2 (− 0.8 to 0.5)	−0.2 (− 0.8 to 0.5)	−0.002 (− 0.6 to 0.6)
Range of motion (degrees)						
Flexion/Extension	1.9 (−1.3 to 5.1)	−0.5 (− 3.7 to 2.7)	0.04 (− 3.2 to 3.3)	1.8 (− 2.8 to 6.4)	−0.5 (− 5.1 to 4.0)	2.4 (− 2.2 to 6.9)
Rotation	− 0.2 (− 3.2 to 2.8)	0.3 (− 2.7 to 3.3)	−0.8 (− 3.8 to 2.2)	0.6 (− 3.7 to 4.9)	1.1 (− 3.1 to 5.4)	−0.5 (− 4.8 to 3.7)
Lateral flexion	− 0.7 (− 3.0 to 1.6)	0.1 (− 2.2 to2.4)	− 2.0 (− 4.3 to 0.3)	1.3 (− 1.9 to 4.6)	2.1 (− 1.1 to 5.3)	−0.8 (− 4.0 to 2.5)

^a^Analysis includes 62 participants in the SMT + HEP group, 61 in the SEP + HEP group, and 61 in the HEP group

(Handgrip strength test was added as an objective measure after the trial was started therefore not available for all participants)

CI, confidence interval; HEP, home exercise program; SEP, supervised exercise program; SMT, spinal manipulative therapy

### Serious adverse events

Six serious adverse events occurred during the trial, all were determined to be unrelated to study interventions. These include: one hospitalization for cholecystectomy and one death due to lung cancer in the HEP group; one hospitalization for acute cardiac symptoms, one new diagnosis of prostate cancer, one injury attending a hockey game, and one transient ischemic attack during follow up phase (not actively in intervention) in the SMT + HEP group; and none in the SEP + HEP group.

## Discussion

### Summary of findings

A limited number of trials have assessed non-pharmacological treatments for the management of low back pain in older adults and to our knowledge, this is the first randomized trial assessing the effectiveness of adding spinal manipulation or a supervised exercise program to a home exercise program in this population. Our primary analysis showed that adding spinal manipulation or a supervised exercise program to a home exercise program did not result in significant or meaningful advantages in pain severity over the course of one year (differences in long-term pain profiles between 0 and 2 percentage points). Secondary analyses showed that at the end of the 12-week treatment phase, group differences favored the addition of SMT or SEP to HEP by 8 and 4 percentage points, respectively. Group differences following the end of treatment were smaller (0 to 3 percentage points) and favored HEP alone over the combined groups. Differences in secondary patient-rated outcomes were small and did not show any clear pattern in favor of one treatment group over another. An exception was satisfaction with care, where participants in both SMT + HEP and SEP + HEP consistently reported greater satisfaction than those in the HEP group throughout the year.

### Clinical importance

A number of factors beyond the magnitude of group differences must be considered when interpreting clinical importance. This includes the magnitude of group differences, proportion of responders, consistency of outcomes, durability of treatment effects, intervention safety and tolerability, treatment adherence, and costs [42]. The magnitude of group differences in pain severity were below the threshold of a moderate effect size (8 percentage points) that was used to power the study, with the exception of SMT + HEP compared to HEP alone at the end of treatment. Responder analyses for reductions in pain severity showed a similar pattern with most group differences falling below 10%. The exception was also at the end of treatment where approximately 15% more participants in the SMT + HEP group reported at least some, 30%, or 50% reductions in pain severity relative to the HEP group. Importantly, following the end of the treatment phase, differences in pain severity favoring SMT + HEP over HEP were *not* maintained and a higher number of participants in the SMT + HEP group sought additional care. In addition, no clear and consistent pattern favoring one treatment over another was noted in key secondary outcomes that would be expected to be similar to pain severity (e.g. disability, improvement). Satisfaction was consistently higher in the combined groups relative to HEP alone. Overall, adherence to treatment regimens was high and no treatment-related serious adverse events occurred. Considering these factors in aggregate, the moderate effect at the end of treatment favoring SMT + HEP over HEP alone should be viewed with great caution. This effect was not durable over time, was not supported by other important secondary outcomes, and may not be clinically important. Differences in healthcare and societal costs are also important factors for assessing clinical importance, and will be reported in a separate publication.

Our rationale for including HEP was that it was a minimal but promising self-management intervention that would be credible to patients [19]. Self-management has been variably defined in the literature, with no commonly accepted definition [14]. Importantly, self-management (and exercise) have become recognized as types of behavior change interventions, which are a coordinated set of activities aimed at changing specified behavior patterns [46]. This is important as the science of behavior change is advancing rapidly with the development of validated reliable taxonomies of behavior change techniques [47–49]. This contemporary understanding allows for the characterization of active ingredients of interventions, like self-management, which may have so far, gone unidentified. This leads us to surmise that our control intervention, HEP, might not have been as inert or minimal as originally anticipated and may explain at least in part, the lack of observed group differences. This issue will be further examined in future analyses.

### Comparison with other trials

The most recent systematic review to address non-pharmacologic treatments for chronic LBP has found evidence of modest short-term improvements in function and/or pain for both exercise and spinal manipulation when compared to usual care, attention control, sham, or placebo, and no clear differences between exercise and spinal manipulation [11]. The results of our study found no difference between SMT + HEP and SEP + HEP and thus appear to confirm the lack of appreciable difference between exercise and SMT as individual modalities. In addition, we found that adding SEP or SMT to HEP does not result in clinically meaningful improvements for older adults with chronic LBP.

A systematic review of manual therapies for older adults found evidence to support its use based on a small number of studies [15]. Not included in that review were three noteworthy studies evaluating SMT for older adults with LBP. The study by Hondras et al. [16] compared two types of SMT (low and high velocity thrust) to medical management in 241 patients 55 years older with subacute and chronic LBP (mean duration more than 12 years). There was no important difference in effectiveness of the two types of SMT, and the low velocity manipulation resulted in small but clinically important short and mid-term improvement in functional status when compared to medical management. Goertz et al. [17] conducted a randomized controlled trial with 131 community-dwelling, ambulatory older adults ages 65 and older with subacute or chronic LBP (84% reporting current LBP episode duration > 1 year). Participants were randomly allocated to 12 weeks of individualized primary medical care (medical care), concurrent medical and chiropractic care consisting primarily of SMT (dual care), or medical and chiropractic care with enhanced interprofessional collaboration (shared care). There were no statistically significant or important differences found between the three groups on the primary measures of low back pain and disability. Finally, a randomized trial by Maiers et al. [50] compared 12 weeks of SMT plus supervised exercise to 36 weeks of the same treatment in 182 adults 65 years of age and older with concurrent low back and neck pain. No significant group differences were observed in low back related disability, the primary outcome measure.

### Strengths and limitations

Our trial has several strengths, including adequate sample size, and a rigorous design with emphasis on internal validity. We also had excellent engagement and follow up rates. Limitations of the study include inability to blind patients and providers to the nature of the interventions. We also did not measure potentially important outcomes or mediators of outcome, including pain catastrophizing, self-efficacy, and therapeutic alliance which are potentially important for older individuals suffering from pain. Also, while we were unable to control for contextual effects which may explain differences in satisfaction, the interventions did approximate what could occur in clinical practice, and are therefore more readily applicable for dissemination.

### Implications for clinical practice

When considered in aggregate, the findings of this study suggest that a four-session home exercise program is a viable treatment option for seniors with chronic LBP. This is supported by the observation that individuals in the HEP group had a similar course of pain over a one-year period to a comparable population of older adults managed in primary medical care [51]. For patients requiring more support, adding spinal manipulation or supervised exercise may be prudent next step.

### Implications for future research

Our findings, especially when considered in light of the cumulative research on non-pharmacologic treatments, including SMT and exercise for low back pain [11, 12] suggest that it may be time to take a step back and re-evaluate how to study this prevalent and costly condition. One such approach may be to learn from the advances in behavioral research focused on the promotion of healthy behaviors, and start focusing more on adaptive pain management behaviors (e.g. more physical activity, self-efficacy and adaptive coping, and less health care utilization, medication use, etc.) and less on the pain symptoms [52]. Further, a more clear delineation of the behavioral components that are likely embedded, but currently unrecognized, in the experimental and control interventions is warranted.

## Conclusion

Adding spinal manipulation or supervised rehabilitative exercise to home exercise alone does not appear to improve pain or disability outcomes in either the short- or long-term in older adults with chronic LBP, but did enhance satisfaction with care. The cost-effectiveness of these interventions needs to be assessed.

## Abbreviations

HEP: Home exercise program
LBP: Low back pain
SEP: Supervised exercise program
SMT: Spinal manipulative therapy
